# Sustained delivery of anti-VEGF from injectable hydrogel systems provides a prolonged decrease of endothelial cell proliferation and angiogenesis *in vitro*

**DOI:** 10.1039/c7ra13014g

**Published:** 2018-02-28

**Authors:** Nathan A. Fletcher, Melissa D. Krebs

**Affiliations:** Department of Chemical and Biological Engineering, Colorado School of Mines 1613 Illinois Street Golden CO 80401 USA mdkrebs@mines.edu

## Abstract

Therapeutic antibodies are attractive treatment options for numerous diseases based on their ability to target and bind to specific proteins or antigens. Bevacizumab, an antiangiogenic antibody, has shown promise for multiple diseases, including various cancers and macular degeneration, where excessive VEGF secretion induces aberrant angiogenesis. In many cases local, sustained delivery of a therapeutic antibody would be preferable to maximize the therapeutic at the disease site, eliminate the need for repeated doses, and reduce systemic side effects. The biodegradable polysaccharides alginate and chitosan can electrostatically interact to form a polyelectrolyte complex (PEC), and have proved effective as a carrier for controlled release of antibodies. In this work, an alginate–chitosan PEC system was designed to produce targeted 30-day delivery of non-specific IgG and anti-VEGF antibodies. The release of anti-VEGF was slow relative to IgG release, suggesting that release rate is antibody specific and is based on the interactions of the PEC with charges present on the antibody surface. The anti-VEGF released from the PEC was shown to successfully inhibit VEGF-induced proliferation and angiogenesis *in vitro* throughout the 30-day test period.

## Abstract 2

Sustained delivery of bioactive anti-VEGF antibodies is demonstrated using a polyelectrolyte complex of alginate and chitosan. The released anti-VEGF inhibited VEGF induced-proliferation and angiogenesis in HUVECs over a 30-day period.

## Graphical abstract

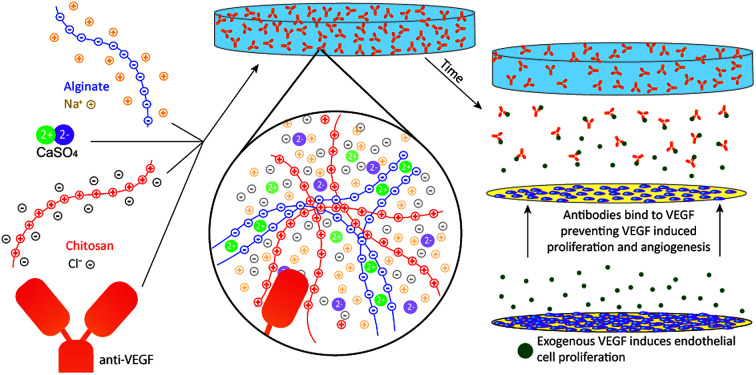

## Introduction

In recent years there has been significant growth in research focused on therapeutic antibodies that can be used to target a wide spectrum of conditions including cancer, chronic inflammatory conditions, transplantation, infectious disease, and cardiovascular diseases.^1^ In 2013, monoclonal antibody sales reached almost $75 billion, which was approximately half of the total global sales of biopharmaceutical products.^2^ As of 2014, 47 monoclonal antibodies had received FDA approval, and with projected growth it is expected there will be 70 monoclonal antibody products in the market by 2020.^2^ Antibodies are large proteins that are a desirable treatment option for many diseases due to their ability to bind to a specific target protein or antigen. While antibodies represent an attractive therapeutic, their commercial success is limited by the high cost of administration due to the need for large and repeated systemic doses, and continued questions of clinical efficacy and potential side effects.^1^ Additionally, increased use of antibody therapies may cause production capacity constraints unless dosing requirements can be decreased or antibody production methods are innovated.^3^ Based on the expected increase in antibody therapy popularity, it is critical to develop delivery strategies for antibody therapeutics that will increase their efficacy and reduce overall required dosing. In many instances, localized delivery of a therapeutic antibody would be advantageous to provide enhanced treatment at the site of interest, reduce systemic side effects, and reduce therapeutic cost by lowering the overall required dosage.

Angiogenesis is the process of creating new capillary blood vessels from pre-existing vasculature. This requires endothelial cells to migrate, proliferate, degrade the basement membrane, and form new lumen.^4^ Vascular endothelial growth factor (VEGF) is an important signalling molecule that instructs endothelial cells to undergo angiogenesis.^5,6^ Glioblastomas and other cancers secrete excess VEGF to induce endothelial cells to undergo angiogenesis at the tumour sites, which increases oxygen and nutrient transport to the tumours.^7,8^ The antiangiogenic antibody bevacizumab (product name Avastin®, Genentech) which targets VEGF is currently approved for treatment of metastatic colorectal cancer and recurrent glioblastoma, and is typically administered by intravenous injection in combination with chemotherapy and radiation.^9,10^ Bevacizumab is also being examined for other clinical applications, including various cancers,^8^ age-related macular degeneration,^11,12^ and to induce cartilage regeneration.^13^

Glioblastoma patients that are administered bevacizumab *via* intravenous injection following tumour resection show improved progression-free survival (PFS) and quality of life, however no improvement in overall survival (OS) time has been shown.^14,15^ The limited efficacy of intravenous bevacizumab treatment for glioblastoma is due, in part, to the blood brain barrier (BBB) which prevents passage of large molecules from the blood stream to the brain tissue and thus to the tumour site.^16^ There have been studies seeking to improve bevacizumab transport across the BBB using selective intra-arterial infusion with BBB disruption, this administration showed an increase in PFS compared to intravenous administration of bevacizumab, but no increase in OS.^17^ Solid lipid nanoparticles (SLNs) have also been studied as a carrier for bevacizumab, the SLNs were shown to be taken up *via* endocytosis by endothelial cells, suggesting they would pass through the BBB.^18^ While techniques to enhance bevacizumab passage across the BBB have been studied, an injectable antibody carrier system that could be placed directly into the brain tissue, thus bypassing the BBB and providing a higher local concentration of therapeutic antibody to the tumour resection site, has yet to be investigated.

Another clinical use of bevacizumab is as a therapy for age-related macular degeneration, where the drug is administered *via* intraocular injection that must be repeated every 4–6 weeks.^19^ Repeated intraocular injections can lead to numerous complications such as intravitreal haemorrhage, endophthalmitis, and retinal detachment; additionally, injection of soluble antibody results in rapid clearance of the therapeutic from the eye.^11,12^ One study compared the injection of soluble bevacizumab to bevacizumab encapsulated in liposomes *in vivo* in a rabbit model, and the liposome-encapsulated bevacizumab was found to be well tolerated with clearance from the eye reduced.^11^ Another study examined the delivery of bevacizumab from blended glycol chitosan and oxidized alginate hydrogels for possible use in ocular drug delivery, but release was complete after 3 days.^19^ Nano and microspheres fabricated from poly(dl-lactide-*co*-glycolide) (PLGA) and poly(ethylene glycol)-*b*-poly(d,l-lactic acid) (PEG-*b*-PLA) have been shown to provide a sustained release of bevacizumab up to 90 days, however harsh solvents are required for the fabrication and antibody activity was not characterized following release.^12^ A thermoresponsive hydrogel of PEG-diacrylate crosslinked poly(*N*-isopropylacrylamide) exhibited sustained release of IgG for 3 weeks and was well tolerated in the rat eye, however this system is not biodegradable.^20^ While numerous systems have been investigated for administration of bevacizumab to the ocular tissue, a biodegradable delivery system with highly controlled sustained release that does not require harsh solvents has yet to be fully realized.

Currently, therapeutic antibodies are delivered intravenously or by subcutaneous injection, this systemic delivery requires large doses to ensure that enough therapeutic antibody reaches the target site, but this also increases the risk of systemic side effects.^21,22^ Intravenous delivery can be particularly ineffective if the target tissue is separated from the blood stream, such as targets in the central nervous system or ocular system. A technology that provides local, sustained delivery of antibodies directly to the target tissue would increase efficacy, reduce dosing requirements, and reduce potential for side effects. There have been some strategies investigated for localized and sustained delivery of antibodies.^22^ Poly(ethylene-*co*-vinyl acetate) (EVAc) has been studied as an implantable antibody release system; it was demonstrated that IgG released from EVAc was present in the brain for up to 28 days compared to a soluble injection where IgG levels dropped within a few days.^23,24^ Although EVAc did provide sustained antibody delivery in the brain, harsh solvents were required to fabricate this system which may impact bioactivity of target-specific antibodies or proteins, and the polymer is not degradable so the implant may require surgical removal following treatment. In another study, IgG antibody was encapsulated in PLGA particles that were dispersed in a blend of hyaluronic acid and methyl cellulose to form an injectable delivery system that displayed antibody release up to 28 days.^25^ Another antibody release system utilized ionic interactions between alginate and monoclonal antibody (mAb1) to provide sustained release over a 10–15 day period with confirmed activity of the antibody after release.^26^ Our group has previously shown that polyelectrolyte complexes (PECs) composed of the polysaccharides alginate and chitosan can be used for delivery of IgG providing sustained release up to 7 weeks.^27^ Antibody release rate can be tailored by altering the polymer ratio of alginate to chitosan or by changing the concentration of CaSO_4_ added to the system.^27,28^ Here, in this work, we tailored the alginate–chitosan PEC delivery system to target a 30-day release of bioactive anti-VEGF antibodies. The bioactivity of the anti-VEGF released from the PECs was confirmed by *in vitro* proliferation and angiogenesis studies using endothelial cells.

## Materials and methods

### Materials

Protanal LF 20/40 alginate and chitosan salt Protasan UP CL 213 (chlorine counterion 13%, batch no. BP-0805-04, 83% deacetylated, 101 mPa s apparent viscosity) were generous gifts from FMC BioPolymer (Philadelphia, PA). A 1 wt% solution of alginate was dialyzed (Fisherbrand, nominal MWCO 3500) for 4 days and subjected to activated charcoal treatment to purify. 1% purified alginate solutions and 1% chitosan salt solutions were sterilized through 0.22 μm filters, frozen and lyophilized. Recombinant human VEGF_165_ was purchased from R&D Systems (Minneapolis, MN). Purified Immunoglobulin G (IgG) derived from human plasma was purchased from Athens Research and Technology (Athens, GA). Anti-human VEGF_165_ monoclonal mouse antibody (anti-VEGF) was obtained from PeproTech (Rocky Hill, New Jersey). Transwell® polyester membranes, 12 mm diameter and pore size of 0.4 μm, were obtained from Corning Incorporated (Corning, NY). Phosphate buffered saline (PBS) with calcium and magnesium was obtained from Gibco (Grand Island, NY). Primary human umbilical vein endothelial cells (HUVECs) from pooled donors, isolated in the absence of defined growth factors without exogenous VEGF, were obtained from Lonza (CC-2519; Basel, Switzerland). CellTracker™ Red CMTPX was obtained from Thermo Fisher Scientific (Waltham, MA). PureCol® Type I bovine collagen was purchased from Advanced BioMatrix (San Diego, CA). Cell Counting Kit-8 (CCK-8) Cell Proliferation and Cytotoxicity Assay was purchased from Dojindo Molecular Technologies (Rockville, MD).

### Antibody release from alginate–chitosan PECs

Alginate–chitosan PECs were formed using solutions of 2 wt% alginate in PBS, 2 wt% chitosan salt in PBS, a calcium sulfate slurry solution (105 mg mL^−1^ in ultrapure water), and either an IgG solution (4.03 mg mL^−1^ in ultrapure water) or an anti-VEGF solution (5 mg mL^−1^ in ultrapure water). PECs were prepared by placing 0.5 mL of alginate solution with 120 μL of ultrapure water (for controls) or 118.6 μL of IgG solution (with 1.4 μL of ultrapure water) or 90 μL of anti-VEGF solution (with 30 μL of ultrapure water) into one 3 mL syringe, and 0.5 mL of chitosan solution with 40 μL of calcium sulfate slurry in a second 3 mL syringe. The two syringes were then connected by a Luer lock adapter and mixed rapidly for 10 seconds. The mixed solution was transferred to a 1 mL syringe and 200 μL aliquots were measured into four Transwell membranes and placed into the center row of a 12-well plate. These studies used 4 replicates (*n* = 4) with final concentrations of 78 μg IgG or 82 μg anti-VEGF in the final PECs. 1 mL of PBS was then added to all wells of the 12-well plate prior to the plate being placed in an incubator (humidified, 37 °C, and 5% CO_2_). Every 5 days, release samples were taken by collecting the PBS from each well and replacing it with fresh PBS. The released antibody in each sample was measured *via* the microBCA protein assay (Pierce, Grand Island, NY) as per the manufacturer's instructions using known quantities of IgG and anti-VEGF for the standard curves.

### Cell culture

Prior to experiments, HUVECs at passage 3 were thawed, seeded, and cultured for 3 days in endothelial growth medium (EGM, CC-3156, Lonza) containing 2% fetal bovine serum (FBS), bovine brain extract, and growth supplements (CC-4133, Lonza) with no exogenous VEGF. Then the medium was replaced with starvation medium (EGM containing 1% FBS and no growth supplements) for 24 hours. The starvation medium was then removed and replaced with 3 mL of 10 μM CellTracker™ Red in EGM and incubated at 37 °C (humidified with 5% CO_2_) for 30 minutes. Cells were then passaged and used for cell proliferation and *in vitro* angiogenesis assays described in later sections.

### Release-conditioned media

Alginate–chitosan PECs with anti-VEGF or water (for controls) were formed by the same procedure as described in the section above (Antibody release from alginate–chitosan PECs). The Transwell membranes containing the PECs were placed in 12-well plates with *n* = 4. Starvation medium was placed in the wells below four of the eight control PECs, while starvation medium with 50 ng of VEGF added was placed in the wells below the four remaining control PECs and the four anti-VEGF PECs. This medium was collected every five days for cell proliferation and *in vitro* angiogenesis assay experiments (described in the following sections), then replaced with fresh medium of the same type.

### Cell proliferation

Every 5 days, 200 μL of conditioned media from each well (*n* = 4 for all three media types, see Release-conditioned media section) was moved to a 48 well plate where the well was coated with type I collagen. On day five 50 μL of starvation medium with 4 × 10^5^ HUVECs per mL were added to each well. After considering the results of day five it was determined that more cells would improve experiment clarity and 50 μL of 8 × 10^5^ HUVECs per mL starvation medium were added to each well for all remaining time points. Cells were then cultured for two days in a humidified incubator at 37 °C and 5% CO_2_. The CCK-8 assay was used to measure cell proliferation, where the reported values are absorbance measurements minus the absorbance of CCK-8 in starvation medium (day five values were normalized by initial seeding density).

### *In vitro* angiogenesis assay

Every 5 days, 600 μL of conditioned medium from each well (*n* = 4 for all three media types, see Release-conditioned media section) was moved to a 48 well plate that contained a 175 μL type I collagen gel that covered the bottom of each well (gels formed per instructions from Advanced Biomatrix, with 8 parts 3 mg mL^−1^ collagen type I, 1 part 10× PBS, then adjusted to pH 7.2–7.4 with 1 part 0.2 M NaOH and ultrapure water). The plates were then placed into the 37 °C incubator for 1 h before removing 400 μL of the conditioned medium. This was done to allow the conditioned medium to diffuse into the type I collagen gels. Then, 50 μL of starvation medium containing 8 × 10^5^ HUVECs per mL were added to each well. Cells were incubated in a humidified environment at 37 °C and 5% CO_2_ for 12 h. At this point the medium from each well was removed and replaced with PBS. Fluorescent images of the cells were taken on a Nikon Eclipse TE 2000S microscope with a Lumenera infinity 3-1UM camera. Total tubule length was calculated using ImageJ software.

### Statistical analysis

Data is expressed as means ± standard deviation. Statistical significance was assessed by two-way ANOVA followed by Turkey's multiple comparison test with *p* < 0.05.

## Results

### Antibody release from alginate–chitosan

**Fig. 1:**
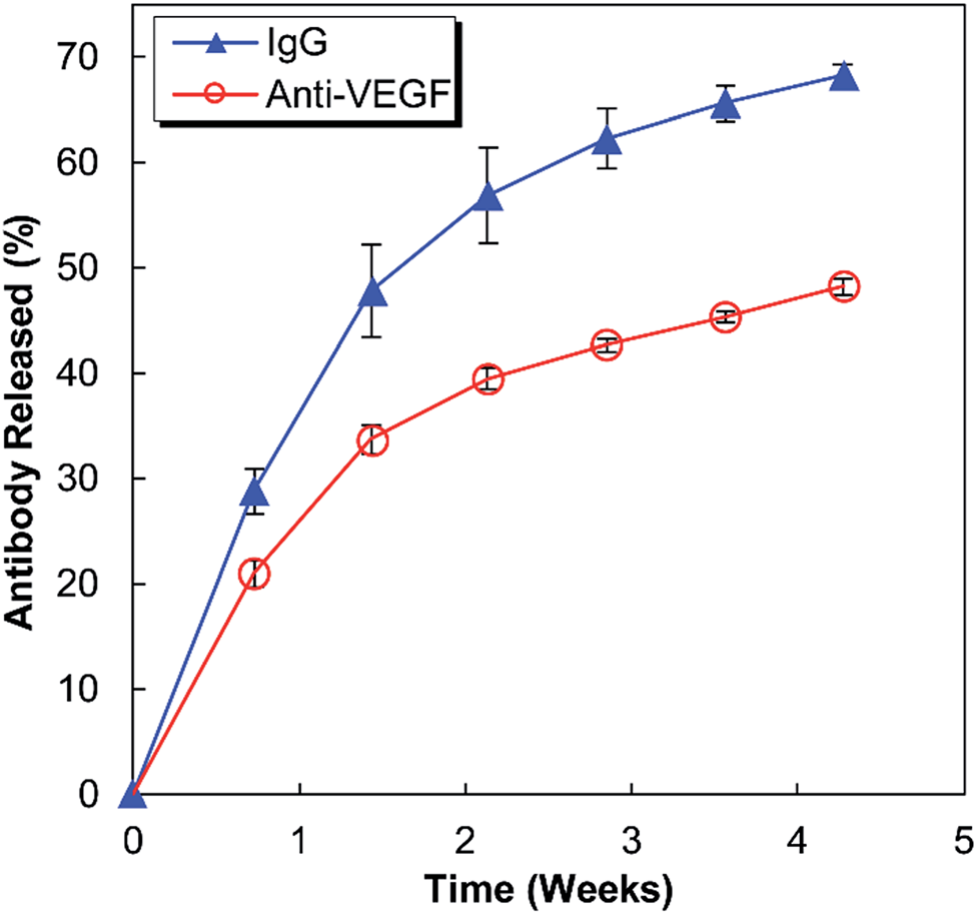
Percent of the total antibody (non-specific IgG or anti-VEGF) loaded in alginate–chitosan PECs that was released over 30 days. The IgG and anti-VEGF release at each timepoint is significantly different (*p* < 0.05).

Anti-human VEGF antibodies (anti-VEGF) and nonspecific IgG antibodies derived from human blood plasma (IgG) were released from separate alginate–chitosan PECs (Fig. 1). Although the release curve trend was similar, the anti-VEGF antibodies were found to release at a reduced rate and quantity compared to the nonspecific IgG antibodies (approximately 70% compared to the IgG at each timepoint). The release is quicker during the first 15 days and then slows, but the antibodies continue to show sustained release out to at least 30 days.

### HUVEC proliferation

**Fig. 2:**
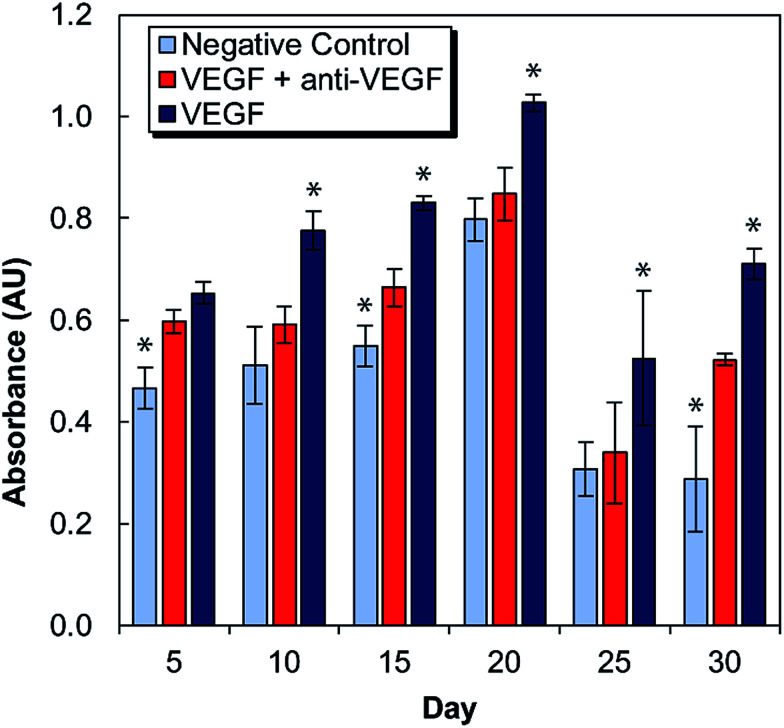
Proliferation of HUVECs in cell culture medium conditioned by exposure to alginate–chitosan PECs every five days over a 30-day period. Light blue bars are the negative controls (medium without VEGF), dark blue are the positive controls (medium with 50 ng mL^−1^ VEGF), both of which were conditioned by blank PECs (without anti-VEGF antibody). The red bars show the experimental group (medium with 50 ng mL^−1^ VEGF) and were exposed to PECs loaded with anti-VEGF antibody. * Indicates a statistically significant difference of the control group from the experimental group with *p* < 0.05.

Medium conditioned by exposure to antibody-laden alginate–chitosan PECs were tested for its effect on HUVEC proliferation every five days for one month (Fig. 2). For these studies, there were two control groups: a negative control of HUVECs cultured in starvation medium exposed to blank alginate–chitosan PECs (without antibody), and a positive control of HUVECs cultured in starvation medium with 50 ng mL^−1^ VEGF added also exposed to blank alginate–chitosan PECs (without antibody). These were compared to the experimental group, which consisted of HUVECs cultured in starvation medium with 50 ng mL^−1^ VEGF added exposed to PECs loaded with anti-VEGF antibody. A statistically significant decrease in proliferation of HUVECs exposed to the anti-VEGF was observed compared to the positive control for all time points out to 30 days except at day 5. Additionally, at days 10, 20, and 25, there is no significant difference between the negative control and the anti-VEGF treated cells, indicating that all VEGF present is bound by the anti-VEGF and unable to influence the cells. Samples from days 10–25 all show the experimental group being closer to the negative control than the positive control.

### *In vitro* angiogenesis assay

**Fig. 3:**
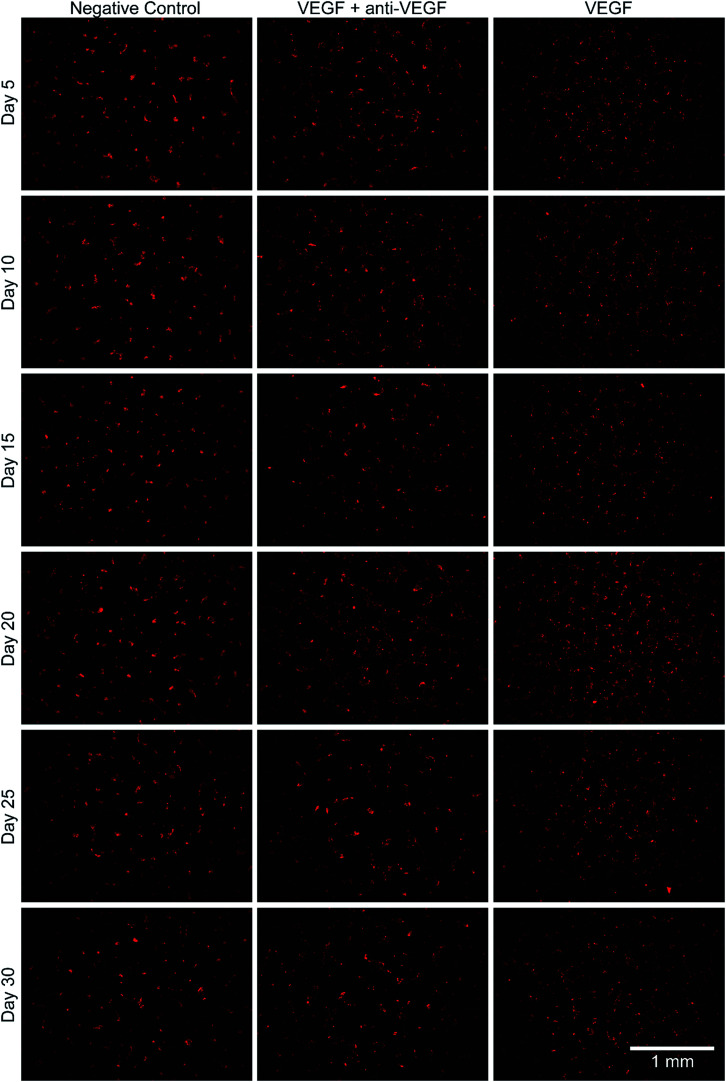
Images of HUVEC *in vitro* angiogenesis after 12 hours of exposure to medium that was conditioned by alginate–chitosan PECs over five day periods, during a 30-day release of anti-VEGF. The media for the negative control group (left images) without VEGF and without anti-VEGF, positive controls (right images) with VEGF but without anti-VEGF, and the experimental group (center images) with VEGF and with anti-VEGF released from the alginate–chitosan PECs used for conditioning.

The bioactivity of the anti-VEGF released from the alginate–chitosan PEC was examined for its ability to prevent VEGF-induced angiogenesis by HUVECs *in vitro* over the course of 30 days. Representative images of HUVECs on type I collagen gels after 12 hours of exposure to the different types of conditioned medium are shown in Fig. 3. All negative controls (without VEGF and without anti-VEGF) show very few cells with minimal connectivity that are only present in small clusters. Positive control samples (with VEGF but without anti-VEGF exposure) have a greater number of cells covering the collagen surface at all timepoints; they also show substantial interconnection between cell clusters that are present throughout the entire field-of-view. The experimental groups that were exposed to both VEGF and released anti-VEGF from the PECs show similar cell populations as the positive controls, however the cell clusters are larger and less interconnected. This indicates that the anti-VEGF released from the PEC is inhibiting the HUVECs' ability to form tubules with one another.

**Fig. 4:**
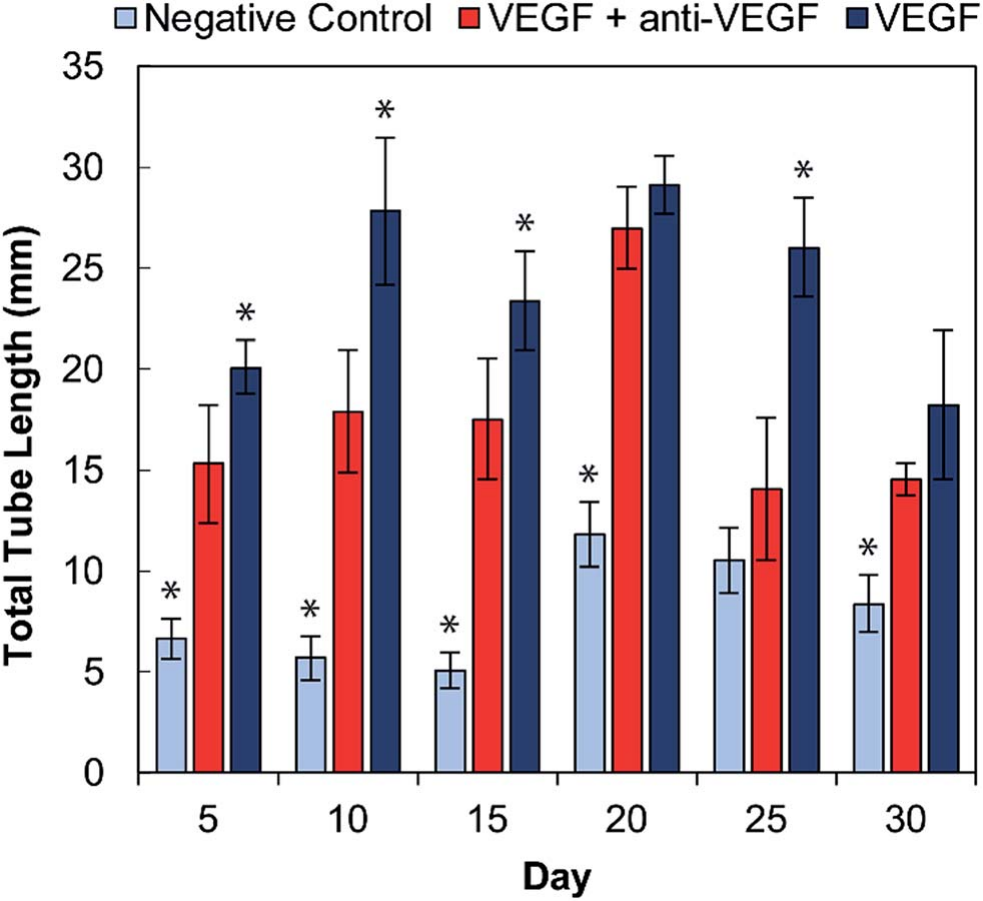
Total tubule formation by HUVECs in cell culture medium conditioned by alginate–chitosan PECs every five days over a 30-day period. Light blue bars are the negative controls (medium without VEGF), dark blue are the positive controls (medium with 50 ng mL^−1^ VEGF), both of which were conditioned by blank PECs (without anti-VEGF antibody). The red bars show the experimental group (medium with 50 ng mL^−1^ VEGF) and were exposed to PECs loaded with anti-VEGF antibody. * Indicates a statistically significant difference of the control group from the experiment group with *p* < 0.05.

The total tubule length (sum of length of all the tubules in the field of view) formed by the HUVECs for each condition was then quantified in ImageJ (Fig. 4). For all time points, the experimental group (with VEGF and with anti-VEGF) show less total tubule formation than the positive control group (with VEGF but without anti-VEGF); and for all days, except days 20 and 30, this reduction in total tubule formation is statistically significant. All days show a significant difference between the experimental group and the negative control except for day 25.

## Discussion

This work investigates the use of alginate–chitosan PECs for the controlled delivery of anti-VEGF antibodies. In previous work, our group has shown that alginate–chitosan PECs can be used to provide sustained release of IgG antibody as a model system, and that the rate of release can be tailored based on the alginate to chitosan ratio.^27^ We have also determined that the addition of CaSO_4_ to the alginate is critical to produce a constant release rate; it is hypothesized that divalent Ca^2+^ ions crosslink some of the alginate leaving unbound chitosan free to electrostatically interact with the antibody, thus providing sustained release.^28^ Based on this previous work, here an alginate : chitosan ratio of 50 : 50 was selected for extended release performance,^27^ and the CaSO_4_ concentration was tailored to target a 30-day antibody release. The release profiles in Fig. 1 display the expected behaviour having a higher initial rate of release, followed by a lower, sustained release out to 30 days. The IgG release is consistently faster compared to the anti-VEGF release; it is hypothesized that the nature of the antibody determines the electrostatic interactions with the chitosan resulting in different release rates. It is well established that antibodies have both positive and negative surface charges and the distribution of these charges will produce a net charge on the antibody. Anti-VEGF (bevacizumab) has been shown to have a pronounced negative effective net charge,^29^ while model IgG is a mixture of antibodies that would have a range of effective net charges.^30^ It is expected that the significant net negative charge results in strong electrostatic interactions between the anti-VEGF and the positively charged chitosan chains, resulting in a slower release rate relative to IgG. Based on successful sustained release of both anti-VEGF and IgG from the alginate–chitosan PECs, we expect this system would be versatile to deliver various or multiple antibodies to a target site, with the knowledge that release rate will be antibody-specific based on the electrostatic interactions of the antibody with the PEC.

VEGF induces proliferation and tube formation of endothelial cells to create new vasculature; this process contributes to the progression of diseases including cancer and macular degeneration. Anti-VEGF antibodies have been demonstrated to inhibit these endothelial cell functions, and anti-VEGF is in clinical use and clinical trials for the treatment of various disease states due to its promise. The ability to provide local, sustained delivery of this antibody over time could further aid in its clinical implementation. It was necessary to ensure bioactivity after encapsulation and maintenance at 37 °C in a buffered solution for a long period of time. Indeed, it was verified that anti-VEGF released from the PECs even out to 30 days could inhibit the increase of proliferation that endothelial cells exhibit in the presence of VEGF. At several of these timepoints, there was no statistical difference between the experimental and negative control groups, indicating that the VEGF-induced proliferation was entirely inhibited.

Endothelial cell tube formation was characterized by a two-dimensional *in vitro* angiogenesis assay. Tube formation can be seen in the fluorescent microscopy images in Fig. 3 and statistical analysis of tube formation can be seen in Fig. 4. Generally, the negative control group, which contained no VEGF in the medium, resulted in a lower number of cells in small clusters with negligible connections to one another, indicating a relatively low level of angiogenesis. The positive control, which had VEGF added to the medium, shows VEGF-induced tube formation as indicated by an increased concentration of cells that were highly interconnected and distributed across the surface. The experimental group, which had VEGF added and was exposed to anti-VEGF from the PEC, had a larger number of cells than the negative control. However, they were growing in large clusters with a lower amount of connections between them compared to the positive control, indicating that VEGF-induced tube formation was partially inhibited. These qualitative claims were confirmed by quantitative image analysis determining the total tubule length found in each image (Fig. 4). Anti-VEGF released from the PECs successfully inhibited the VEGF-induced tube formation, as indicated by the experimental group falling between the negative and positive controls for total tubule length at all time points. The reduction in total tubule length of the experimental group compared to the positive control is statistically significant at all time points, except for days 20 and 30, indicating that the anti-VEGF released from the PEC successfully binds soluble VEGF present in the medium to inhibit tube formation. For day 20, the total tubule length of the experimental group nearly reaches that of the positive control group, we expect that this is caused by an increased cell seeding density for day 20 experiments; this is supported by increased values in the proliferation data (Fig. 2). Through qualitative observation, it is expected that the increased cell seeding density produced overlapping cell clusters which were quantified as increased tubule length but do not demonstrate true connectivity like that seen in the positive control group. Based on 2-D angiogenesis images and quantitative analysis of tubule formation length, anti-VEGF release from alginate–chitosan PECs can successfully inhibit VEGF-induced angiogenesis of HUVECs *in vitro*.

## Conclusions

In this work, we demonstrated sustained delivery of bioactive anti-VEGF antibodies using an alginate–chitosan PEC for injectable delivery to therapeutic target locations. This system is capable of delivering multiple antibodies, with release rate that can be antibody-specific. Sustained delivery of anti-VEGF antibody was demonstrated over a 30-day period. The anti-VEGF released from the PECs was shown to successfully inhibit VEGF-induced proliferation and angiogenesis of HUVECs *in vitro* for the entire 30-day period. VEGF is an important signalling protein for angiogenesis, which is involved in multiple disease progressions, therefore blocking this signalling *via* a local, controlled delivery technology could have significant clinical benefit.

## Conflicts of interest

There are no conflicts to declare.

## Supplementary Material
