# Correction: Sustained delivery of anti-VEGF from injectable hydrogel systems provides a prolonged decrease of endothelial cell proliferation and angiogenesis *in vitro*

**DOI:** 10.1039/c8ra90033g

**Published:** 2018-05-01

**Authors:** Nathan A. Fletcher, Melissa D. Krebs

**Affiliations:** Department of Chemical and Biological Engineering, Colorado School of Mines 1613 Illinois Street Golden CO 80401 USA mdkrebs@mines.edu

## Abstract

Correction for ‘Sustained delivery of anti-VEGF from injectable hydrogel systems provides a prolonged decrease of endothelial cell proliferation and angiogenesis *in vitro*’ by Nathan A. Fletcher *et al.*, *RSC Adv.*, 2018, **8**, 8999–9005.

One of the funding sources (NIH) was inadvertently omitted in the published article; the corrected acknowledgements section is shown below.

The authors gratefully acknowledge funding support from the Boettcher Foundation (#11219) and the National Institute of Arthritis and Musculoskeletal and Skin Diseases of the National Institutes of Health (#1R03AR068087). The content is solely the responsibility of the authors and does not necessarily represent the official views of the National Institutes of Health or the Boettcher Foundation.

The Royal Society of Chemistry apologises for these errors and any consequent inconvenience to authors and readers.

## Supplementary Material

